# Polarimetric parity-time symmetry in a photonic system

**DOI:** 10.1038/s41377-020-00407-3

**Published:** 2020-09-27

**Authors:** Lingzhi Li, Yuan Cao, Yanyan Zhi, Jiejun Zhang, Yuting Zou, Xinhuan Feng, Bai-Ou Guan, Jianping Yao

**Affiliations:** 1 Guangdong Provincial Key Laboratory of Optical Fiber Sensing and Communications, Institute of Photonics Technology, Jinan University, Guangzhou, 511442 China; 2 Microwave Photonics Research Laboratory, School of Electrical Engineering and Computer Science, University of Ottawa, Ottawa, ON K1N 6N5 Canada

**Keywords:** Fibre lasers, Optical techniques

## Abstract

Parity-time (PT) symmetry has attracted intensive research interest in recent years. PT symmetry is conventionally implemented between two spatially distributed subspaces with identical localized eigenfrequencies and complementary gain and loss coefficients. The implementation is complicated. In this paper, we propose and demonstrate that PT symmetry can be implemented between two subspaces in a single spatial unit based on optical polarimetric diversity. By controlling the polarization states of light in the single spatial unit, the localized eigenfrequencies, gain, loss, and coupling coefficients of two polarimetric loops can be tuned, leading to PT symmetry breaking. As a demonstration, a fiber ring laser based on this concept supporting stable and single-mode lasing without using an ultranarrow bandpass filter is implemented.

## Parity-time symmetry: Achieving single-mode lasing in long cavities

Parity-time symmetry can be implemented between two sub-spaces in a single long cavity to achieve stable single-mode lasing without needing expensive optical filters. Jiejun Zhang and colleagues at China’s Jinan University demonstrated this by manipulating linearly and orthogonally polarized light waves in a single-loop fiber-ring laser. They adjusted the gain, loss and coupling between the polarized light waves, to ensure that the system operates with parity-time symmetry breaking. This generated a single-mode light wave that inherently covers a narrow part of the electromagnetic spectrum (2.4 kHz), but has a larger measured ‘linewidth’ of 129 kHz, due to the long cavity’s susceptibility to environmental disturbances. This linewidth could be reduced by actively stabilizing or isolating the cavity. The single physical loop design provides simplicity and highly improved stability to the system.

## Introduction

A parity-time (PT) symmetric system is a special non-Hermitian system of which its Hamiltonian possesses real eigenvalues. Recently, PT symmetry has been investigated extensively in photonic^1–13^ and optoelectronic^14,15^ systems, one of the main reasons being its effectiveness for mode selection in an optical or optoelectronic system^11–15^. For a high-performance continuous-wave laser, single-mode operation is one of the fundamental requirements because it determines the coherence and power stability of the laser output^5,6,16^. For a long-cavity laser, for which it is preferable to have low phase noise, a narrow linewidth, and high optical power, single-mode lasing is challenging due to the small mode spacing^5,6,16–24^. Since the gain spectrum of a gain medium in a laser cavity is usually several orders of magnitude broader than the mode spacing of the laser, the round-trip gain difference between adjacent modes is small, making it difficult to achieve single-mode oscillation by manipulating the lasing threshold^5,6^. An optical filter can be incorporated into a laser cavity to reduce the number of modes above the threshold. If the optical filter has a sufficiently narrow passband, single-mode lasing can be achieved^23,24^. However, for a long-cavity laser such as a fiber laser with a cavity length on the order of tens of meters, a high-Q optical filter is needed, making the system costly and endowing it with poor stability^25,26^.

Parity-time symmetry has been proven to be an effective solution for achieving mode selection in a photonic system^1–15^. Conventionally, a PT-symmetric system is implemented between two cross-coupled and spatially distributed optical subspaces, which are engineered to have an identical geometry with complementary gain and loss coefficients. A PT-symmetric system has a strongly enhanced gain difference between the dominant mode and the side modes, thus making single-mode oscillation possible^5,6,14,15^. However, such a system requires two spatially distributed subspaces, which leads to increased structural complexity, high cost, and strong susceptibility to environmental perturbations. Recently, we demonstrated a new type of PT-symmetric system that is realized in the parameter space of optical wavelength, where the spatial duplicity in a conventional PT-symmetric system is eliminated^27^. Since two wavelengths are employed, the concept is not applicable to a system where only a single wavelength is supported.

In this paper, we propose and demonstrate that a PT-symmetric system can be implemented in a single spatial unit based on polarimetric diversity, which supports single-wavelength operation. By controlling the polarization states of light in a single spatial unit, the localized eigenfrequencies, gain, loss, and coupling coefficients can be controlled to achieve PT symmetry breaking. As a demonstration, a long-cavity fiber ring laser supporting single-mode lasing without using a high-Q optical filter is implemented. In the experiment, the fiber ring laser has a cavity length of 41.0 m with a mode spacing as small as 4.88 MHz. The employment of polarimetric PT symmetry enables effective suppression of the sidemodes with a suppression ratio greater than 47.9 dB. The linewidth of the light generated by the fiber ring laser is measured to be 129 kHz with a wavelength tunable range of 35 nm. With active stabilization, the linewidth can be reduced to its Lorentzian linewidth of 2.4 kHz. The proposed polarimetric PT symmetry concept opens new avenues for the implementation of non-Hermitian photonic systems with increased functionalities.

## Results

**Fig. 1:**
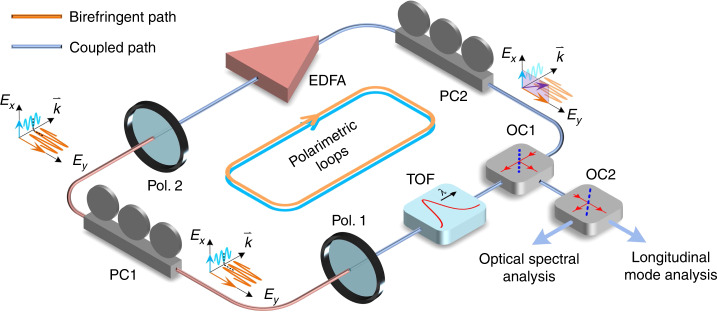
Schematic diagram of the proposed polarimetric PT-symmetric photonic system. The system consists of a single spatial loop, in which two equivalent polarimetric loops are formed by recirculating light waves of orthogonal polarization states in the loop. To achieve PT symmetry, the phase retardance, power ratio, and coupling coefficient between the orthogonally polarized light waves are tuned by controlling PC1 in the birefringent path, and the lasing threshold is tuned by controlling PC2 in the coupled path. PC: polarization controller; Pol.: polarizer; EDFA: erbium-doped fiber amplifier; OC: optical coupler; TOF: tunable optical filter

Figure 1 shows a schematic diagram of the proposed polarimetric PT-symmetric photonic system consisting of two polarimetric loops. The two polarimetric loops, with independently adjustable eigenfrequencies and round-trip gain coefficients, are implemented based on polarimetric diversity in a single physical loop. Specifically, the birefringent path in the fiber ring laser loop creates two polarimetric loops, and the coupled path allows coupling between the two polarimetric loops. Two polarization controllers (PCs), in conjunction with two polarizers (Pol. 1 and Pol. 2), are used to tune the gain and loss coefficients of the eigenvalues in the two polarimetric loops to achieve PT symmetry. As the gain and loss coefficients are increased to values greater than the coupling coefficient, PT symmetry breaking occurs. The two polarimetric loops form a PT-symmetric fiber ring laser with one polarimetric loop supporting the gain modes and the other supporting the loss modes. The polarimetric PT symmetry then strongly enhances the gain difference between the dominant mode and the sidemodes, making single-mode lasing possible without using an ultranarrow bandpass filter.

As shown in Fig. 1, the photonic system has a single unidirectional physical fiber loop that supports two polarimetric loops. An erbium-doped fiber amplifier (EDFA) is incorporated to provide an optical gain. The polarimetric diversity is implemented by controlling the polarization states of light in the fiber loop. The dominant mode can be coarsely chosen with a tunable optical filter (TOF). The output of the system is derived using a 3-dB optical coupler (OC), which is sent to an optical spectrum analyzer (OSA) for spectrum analysis, an optical homodyne system for mode analysis, and an optical self-heterodyne system for linewidth measurement.

A three-paddle fiber-optic PC has a sandwiched structure with a half-wave plate located between two quarter-wave plates. The transfer function of a three-paddle PC is given by^28^

1 $$F_{PC} = \left[ {\begin{array}{*{20}{c}} {\exp \left( {i\varphi _2} \right)} & 0 \\ 0 & 1 \end{array}} \right] \times \left[ {\begin{array}{*{20}{c}} {\cos \theta } & { - \sin \theta } \\ {\sin \theta } & {\cos \theta } \end{array}} \right] \times \left[ {\begin{array}{*{20}{c}} {\exp \left( {i\varphi _1} \right)} & 0 \\ 0 & 1 \end{array}} \right]$$

where *φ*_1_ and *φ*_2_ are the phase retardances introduced to the two orthogonally polarized light waves by the two quarter-wave plates and *θ* is the rotation angle of the polarization direction introduced by the half-wave plate. As can be seen, a PC can introduce independent polarization direction rotation and polarization phase retardance to an incident light. In our system, polarimetric PT symmetry is achieved by controlling three critical angles in PC1 and PC2, which are listed in Table 1.

**Table 1:** The critical angles that are tuned to achieve polarimetric PT symmetry

Angle	Definition	Functionality	Tuning method
*θ*_*r*_	Rotation angle of quarter-wave plate to tune polarization phase retardance	PT symmetry of the real part of the eigenfrequency of the polarimetric loops	Quarter-wave plate of PC1
*θ*_*i*_	Optical axis rotation angle from Pol. 1 to Pol. 2	PT symmetry of the imaginary part of the eigenfrequency of the polarimetric loops	Half-wave plate of PC1
*θ*_*t*_	Optical axis rotation angle from Pol. 2 to Pol. 1	Lasing threshold	Half-wave plate of PC2

**Fig. 2:**
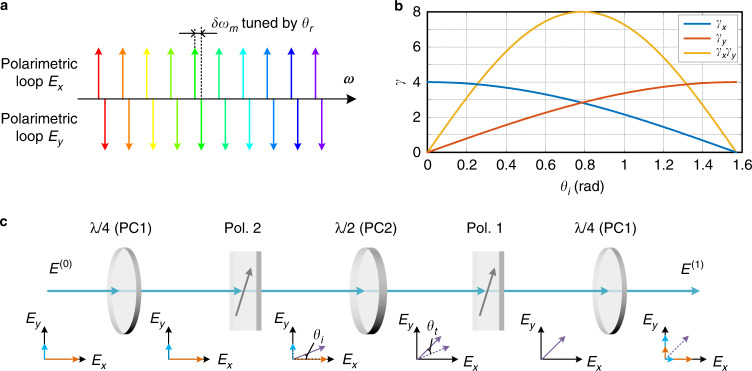
Polarimetric PT symmetry achieved by controlling the polarizations by tuning PC1 and PC2 in the photonic system. **a** Compensation for eigenfrequency separation between the polarimetric loops when the phase retardances between *E*_*x*_ and *E*_*y*_ are tuned by the quarter-wave plate of PC1; **b** round-trip gain and loss coefficient variations of the polarimetric loops when the polarization directions of *E*_*x*_ and *E*_*y*_ are tuned by the half-waveplate of PC1; and **c** illustration of the gain, loss and coupling of the polarization components as light at PC1 propagates through Pol. 1, PC2, and Pol. 2 and then returns to PC1. Part of the polarization component *E*_*x*_ is coupled to the other polarization component *E*_*y*_, and vice versa

Specifically, the bending-induced birefringence of PC1 leads to a polarization phase retardance. Assuming that the fast and slow polarization components are *E*_*x*_ and *E*_*y*_, respectively, the phase retardance can be tuned by changing *θ*_*r*_ using the equivalent quarter-wave plates in PC1, which aligns the eigenmodes of the two polarimetric loops. The *m*-th order eigenmode of a loop resonator is given by

2 $$\omega _m = \frac{{\left( {2m\pi + \varphi } \right)c}}{{n_{eff}L}}$$

where *φ* is the phase shift within the laser ring cavity, *c* is the light velocity in a vacuum, and *n*_eff_ and *L* are the effective refractive index and the length of the optical fiber within the laser ring cavity, respectively. Although the physical lengths of the two polarimetric loops are the same, the eigenfrequencies of the cavities with the respective polarizations may be perturbed due to the residual birefringence originating from the fiber bending and the polarization mode dispersion (PMD) of the optical components, resulting in a mismatch of the eigenmodes between the two polarimetric cavities, as shown in Fig. 2a. Assuming that the birefringent phases of the two polarizations are *φ*_*x*_ and *φ*_*y*_, the separation between the localized eigenfrequencies is given by

3 $$\delta \omega _m = \frac{{( {\varphi _x - \varphi _y})c}}{{n_{eff}L}}$$

To achieve PT symmetry, it is required that the eigenfrequency separation is zero, which can be achieved by tuning the equivalent quarter-wave plate in PC1, i.e., *θ*_*r*_, to match the values of *φ*_*x*_ and *φ*_*y*_, thus aligning the eigenfrequencies of the polarimetric cavities for the implementation of PT symmetry between the real parts of the eigenfrequencies^29^.

On the other hand, the tuning of *θ*_*i*_ using the half-wave plate in PC1 can result in a rotation of the optical axis in the optical path from Pol. 1 to Pol. 2, which, in conjunction with the polarizers, adjusts the round-trip gain, loss, and coupling coefficients of the two polarimetric loops. PT symmetry between the imaginary parts of the eigenfrequencies of the two polarimetric loops can be achieved. We first assume that the polarization direction of the light is preserved from Pol. 2 to Pol. 1, i.e., *θ*_*t*_ = 0, and that the directions of Pol. 1 and Pol. 2 are aligned such that they are the same. The joint operation between Pol. 1 and Pol. 2 is then equivalent to a single polarizer. The relations between the electric fields at PC1 before and after one round trip in the cavity, denoted as *E*^(0)^ and *E*^(1)^, are given by

4 $$\left[ {\begin{array}{*{20}{c}} {E_x^{\left( 1 \right)}} \\ {E_y^{\left( 1 \right)}} \end{array}} \right] = \gamma _0\left[ {\begin{array}{*{20}{c}} {{\cos}^2\theta _i} & {\sin \theta _i\cos \theta _i} \\ {\sin \theta _i\cos \theta _i} & {{\sin}^2\theta _i} \end{array}} \right]\left[ {\begin{array}{*{20}{c}} {E_x^{\left( 0 \right)}} \\ {E_y^{\left( 0 \right)}} \end{array}} \right]$$

where *γ*_0_ is the round-trip gain of the electric field of a polarization component when its polarization direction is perfectly aligned with that of the polarizer.

As the polarization components *E*_*x*_ and *E*_*y*_ recirculate in the fiber loop, their incidental angles respective to the principal axes of Pols. 1 and 2 result in round-trip gains, given by

5 $$\gamma _x = \gamma _0{\cos}^2\theta _i$$

6 $$\gamma _y = \gamma _0{\sin}^2\theta _i$$

and a round-trip coupling strength given by

7 $$\kappa = \gamma _0\sin \theta _i\cos \theta _i$$

The round-trip gains *γ*_*x*_ and *γ*_*y*_ can be tuned continuously from 0 to a maximum value of *γ*_0_. At PT symmetry, gain and loss balance should be achieved between the two polarimetric loops, i.e., *γ*_*x*_*γ*_*y*_ = 1. Based on Eq. (5), we find that the angle of polarization rotation for the implementation of PT symmetry for the imaginary part of the eigenfrequency is

8 $$\theta _\gamma = \pm \frac{1}{2}\arcsin \left( {\frac{2}{{\gamma _0}}} \right)$$

Last, the optical path from Pol. 2 to Pol. 1 is initially configured to be nonbirefringent by tuning PC2 to fully compensate for the polarization mode dispersion contributed by all optical components. The tuning of the equivalent half-wave plate in PC2 then introduces a propagation loss for any incident light to Pol. 1, as the polarization direction of light from Pol. 1 is no longer perfectly aligned with that of Pol. 2. The maximum net round-trip gain becomes *γ*_0_cos*θ*_*t*_ instead of *γ*_0_, where *θ*_*t*_ is the polarization rotation angle. Tuning *θ*_*t*_ is different from the case where *θ*_*i*_ is tuned. The tuning of *θ*_*i*_ introduces a variation in the gain difference between the polarimetric loops and can be used to achieve gain and loss balance. By contrast, the tuning of *θ*_*t*_ introduces a universal gain variation of cos*θ*_*t*_ for both polarimetric loops and thus affects only the lasing threshold of the PT-symmetric laser. Tuning *θ*_*t*_ is an alternative way to adjust the lasing threshold, which is more precise and convenient than changing the pump current to the EDFA.

We then convert the round-trip gain, loss and coupling to the corresponding per-time-unit coefficients (see Supplementary Information 1), and the optical coupling between the two polarimetric loops can be written as (see Supplementary Information 2)

9 $$\frac{{dE_x}}{{dt}} = - i\omega _mE_x + i\kappa E_y + \gamma _xE_x$$

10 $$\frac{{dE_y}}{{dt}} = - i\omega _mE_y + i\kappa E_x + \gamma _yE_y$$

where *κ* is a real number^30^. The solution to the coupling equations shows that a PT-symmetric system has complex eigenfrequencies when the gain or loss coefficients have a higher magnitude than the coupling coefficient, in which the imaginary part represents the gain or loss coefficient of the mode^14^. When operating in PT symmetry, the system can provide a gain difference enhancement between the longitudinal mode with the highest round-trip gain (dominant mode, *γ*_0_) and that with the second highest gain (secondary mode, *γ*_1_). The enhancement factor is given by

11 $$F = \frac{{{\Delta} g_{PTS}}}{{{\Delta} g_{Hermitian}}} = \frac{{\sqrt {\gamma _0^2 - \gamma _1^2} }}{{\gamma _0 - \gamma _1}}$$

where Δ*g*_*PTS*_ and Δ*g*_*Hermitian*_ are the gain differences between the dominant and secondary modes in a PT-symmetric system and a single-loop Hermitian system, respectively. The enhancement of the gain difference with a factor of *F* would significantly reduce the difficulty in achieving stable single-mode lasing.

## Discussion

**Fig. 3:**
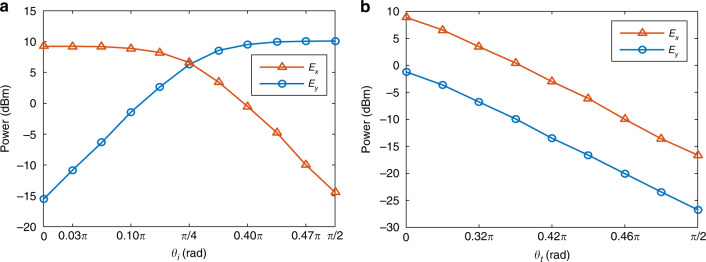
Gain tuning in the two polarimetric loops achieved by tuning PC1 and PC2. The round-trip gain coefficients of the two polarimetric loops change **a** differentially and **b** uniformly when *θ*_*i*_ and *θ*_*t*_ are tuned, respectively. The measurement results agree well with the theoretical results shown in Fig. 2b, indicating that PT symmetry can be achieved by tuning PC1 and PC2

An experiment is performed based on the setup shown in Fig. 1. Figure 3 shows the round-trip gain variations of the polarimetric loops measured by injecting two linearly and orthogonally polarized light waves into the open-loop laser cavity. By tuning PC1 and PC2, the round-trip gain and gain difference between the two polarimetric loops are changed, which can be used to implement PT symmetry in the proposed polarimetric PT-symmetric fiber ring laser (see Supplementary Information 3).

**Fig. 4:**
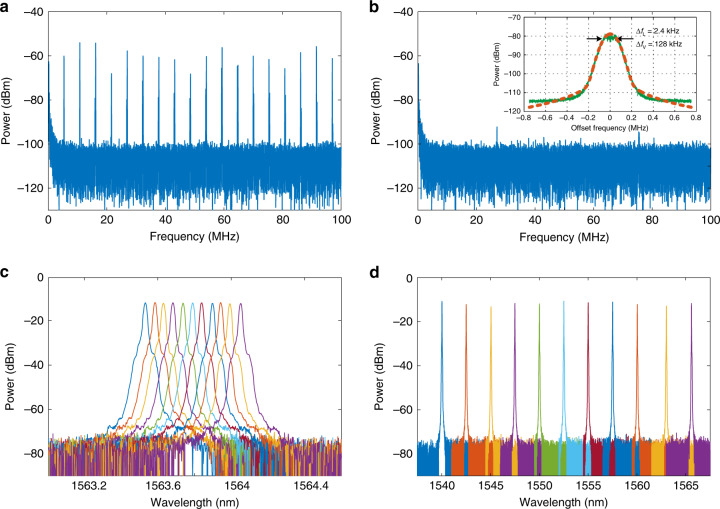
Spectrum analysis. **a** The homodyne spectrum when the system is operating without PT symmetry. Multimode oscillation is observed. **b** The homodyne spectrum when the system is operating with PT symmetry by tuning PC1 and PC2. Single-mode oscillation is observed. The inset shows that the laser linewidth is measured to be 129 kHz, which is broadened due to the environmental perturbations in the long-cavity laser. The Lorentzian linewidth is calculated to be 2.4 kHz. **c**, **d** The optical spectra of the single-mode laser when the wavelength is **c** fine-tuned with a step of 50 pm from 1563.55 to 1564.05 nm and **d** coarsely tuned with a step of 2.5 nm from 1540 to 1565 nm

To verify the PT symmetry operation of the fiber ring laser, the light at its output is directed to an optical spectrum analyzer for spectrum measurement, a homodyne system for longitudinal mode analysis, and a self-heterodyne system for spectral linewidth measurement. When the loop is closed and the EDFA operates with a sufficiently high gain to make the round-trip net gain exceed the loss, lasing begins. Figure 4a shows the homodyne spectrum. Since PC1 and PC2 are not tuned to achieve PT symmetry, multimode lasing with a mode spacing of 4.88 MHz corresponding to a loop length of 41.0 m is observed from the measured electrical spectrum. As PC1 and PC2 are tuned to achieve PT symmetry, and to make the gain/loss greater than the coupling coefficient, PT symmetry is broken, and single-mode lasing is achieved. As can be seen in Fig. 4b, all the beat notes are suppressed except the one at DC, which clearly indicates that single-longitudinal-mode lasing is achieved. The measured optical spectrum is shown in Fig. 4c with a central wavelength of 1563.204 nm. The highest beat notes from Fig. 4a, b are suppressed by 47.9 dB compared to the DC, which indicates that a sidemode suppression ratio of approximately 47.9 dB is achieved due to the PT symmetry. The self-heterodyne spectrum is shown in the inset of Fig. 4b. A linewidth of approximately 129 kHz is measured. By fitting the self-heterodyne spectrum to the Voigt profile^31^, we find that the Lorentzian linewidth Δ*f*_*L*_ and the Voigt linewidth Δ*f*_*V*_ are 2.4 and 128 kHz, respectively, which indicates that the light wave generated by the fiber ring laser has a very narrow intrinsic linewidth of 2.4 kHz thanks to the long cavity length. On the other hand, the measured linewidth is 129 kHz, which is broadened due to the high susceptibility of the system to environmental disturbances because of a long fiber employed in the cavity. This broadening can be well compensated if active cavity stabilization or isolation is incorporated in the laser system^31,32^.

In addition, the power stability is also measured. As shown in the supplemental video, the power fluctuation is as low as 0.2 dB within a 30-s measurement time frame. If the system is well packaged or active cavity stabilization or isolation is incorporated, the power stability can be further improved.

Then, the wavelength tunability of the fiber ring laser is studied. Figure 4c, d show the optical spectra of the light at the output of the fiber ring laser when the wavelength is tuned with a minimum tuning step of 50 pm and a maximum tuning range of 25 nm, from 1540 to 1565 nm. This is achieved by tuning the TOF, which has a bandwidth of 0.1 nm, within which there are approximately 2016 longitudinal modes. PT symmetry is then realized to help select the longitudinal mode with the highest round-trip gain from the 2,016 longitudinal modes to enable stable single-mode lasing.

**Fig. 5:**
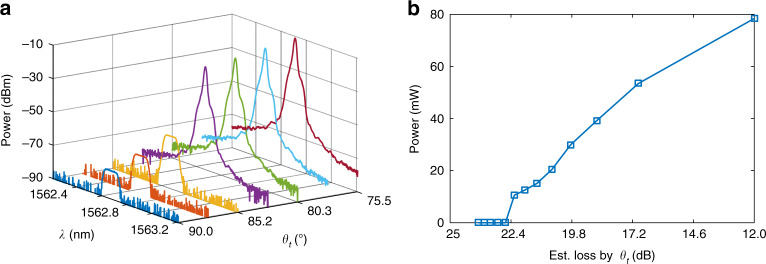
Optical spectrum and output power of the fiber ring laser when the cavity loss is tuned by tuning PC2. **a** Optical spectrum evolution of the laser output when the intracavity loss is tuned by tuning PC2, showing the threshold-like behavior of the polarimetrically PT-symmetric laser. **b** Output optical power measured from the optical spectrum at peak wavelength when the intracavity loss is tuned within a range of 10 dB. Note that an optical attenuator is incorporated when the optical spectrum is measured to avoid high-power damage to the OSA

The threshold behavior of the fiber ring laser is also studied. Figure 5a shows the optical spectrum at the output of the fiber ring laser. As shown, when the gain is below the threshold, the fiber ring laser is in the nonlasing mode, and a broad spectrum, which is the filtered amplified spontaneous emission (ASE), is produced. When the gain is sufficiently high as a result of reducing the intracavity loss by tuning PC2, lasing starts, and a high-power optical output at 1562.712 nm is observed. Figure 5b shows the output power when the intracavity loss is tuned.

In conclusion, we have proposed and demonstrated that PT symmetry can be implemented between two subspaces in a single spatial unit based on polarimetric diversity. By controlling the polarization states of light in the single spatial unit, the localized eigenfrequencies, gain, loss, and coupling coefficients could be tuned, leading to PT symmetry breaking. The polarimetric PT symmetry was experimentally verified by a fiber ring laser in which a single physical loop supporting two mutually coupled polarimetric loops was implemented. Stable single-longitudinal-mode lasing was achieved. The linewidth of the light generated by the laser was measured to be 129 kHz for a wavelength tuning range of 35 nm. With active stabilization implemented in the fiber laser, the linewidth could be reduced to its Lorentzian linewidth of 2.4 kHz. Since only a single physical loop is required, the implementation is significantly simplified, and the stability is highly improved.

## Materials and methods

The polarimetric PT symmetry is demonstrated based on a fiber ring laser implemented using commercial off-the-shelf optical components. The EDFA (Amonics AEDFA-27-B-FA) has an operating wavelength range from 1535 to 1565 nm and a fixed output power of 27 dBm. The TOF (Alnair Labs BVF-300CL) has a tunable 3-dB bandwidth from 0.03 to 3 nm and a tunable central wavelength from 1525 to 1610 nm. The PCs are Thorlabs FPC032 manual paddle fiber polarization controllers. The polarizers are implemented with a polarization beam combiner/splitter (Thorlabs PBC1550SM-APC) with only two reciprocally transmitting ports.

The performance of the system is evaluated using benchtop instruments. The optical spectra are measured with an OSA (Yokogawa AQ6370C). An optical homodyne is used for laser modal analysis due to its high dynamic range, which is realized by directly launching the light at the output of the laser to a PD (photodetector, Discovery Semiconductor DSC20H) and measuring the electrical spectrum at the output of the PD with an ESA (electrical spectrum analyzer, Keysight N9020B). The linewidth of the light wave generated by the laser is measured based on the optical self-heterodyne, in which a 40-km-long single-mode fiber is used to introduce a long time delay and an intensity modulator (Photline MX-LN-40), biased at the minimum transmission point driven by a microwave source (HMC-T2220), is used to slightly shift the wavelength to make the beat note easier to observe. The optical self-heterodyne spectrum is also measured with a PD (Discovery Semiconductor DSC20H) and an ESA (Keysight N9020B).

## Supplementary information

Supplementary Information for Polarimetric parity-time symmetry in a photonic system

The real-time optical and homodyne electrical spectra
