# Supplementary material for: Polarimetric parity-time symmetry in a photonic system
Source: Light Sci Appl. 2020 Sep 27;9:169. doi: 10.1038/s41377-020-00407-3 (PMC7520423; doi:10.1038/s41377-020-00407-3)
Supplement: Supplementary file 1 — Supplementary Information for Polarimetric parity-time symmetry in a photonic system [file 41377_2020_407_MOESM1_ESM.docx]

Supplementary Information for
*Polarimetric parity-time symmetry in a photonic system*

**Authors:** Lingzhi Li, Yuan Cao, Yanyan Zhi, Jiejun Zhang*, Yuting Zou, Xinhuan Feng,
Bai-Ou Guan and Jianping Yao*

**1. Roundtrip gain, loss and coupling and corresponding time-based coefficients**

In a resonator-based system, the magnitude of gain, loss and coupling can be represented either in a per roundtrip notation or in a per time-unit notation. In our manuscript, both notations are used. The differences are discussed below.

The evolution of the electric field in a laser cavity can be described by

$$E\left(t\right)=E\left(0\right)e^{\frac{\gamma}{T_{r}}t}$$

where $E\left(0\right)$ is the initial electric field, $\gamma/T_{r}$ is the gain coefficient, and $T_{r}$ is the round-trip time. The electrical field after one round trip is $E\left(T_{r}\right)=E\left(0\right)e^{\gamma}$. It can be seen that $E\left(T_{r}\right)/E\left(0\right)=e^{\gamma}$ is the roundtrip gain which is the ratio between the electrical fields after and before recirculating in the resonator for one round-trip. The corresponding gain coefficient can is given by $\gamma/T_{r}=\left[\ln E\left(T_{r}\right)-\ln E\left(0\right)\right]/T_{r}$. Similarly, the round-trip loss and coupling can be converted to the corresponding loss and coupling coefficients.

In our analysis, the gain, loss and coupling introduced by the polarizers and the polarization controllers are per-roundtrip values. On the contrary, those quantities are more conveniently expressed as time-based coefficients in the coupled differential equations. The per roundtrip notation and per time-unit notation are not explicitly stated in the main text, but they can be easily differentiated by observing how the quantities are used.

**2. Coupling between polarimetric loops**

Figure S1(a) shows a spatial PT-symmetric system that is equivalent to the proposed polarimetric PT-symmetric system we demonstrated, in which the coupling between the polarization modes using polarizers and polarization controllers are implemented by a lossless coupler in conjunction with four attenuators (Atts. 1, 2 and 3s). Fig. S1(b) shows the transfer matrix of the passive and lossless coupler. It should be noted that, in our actual system, the EDFA is located in the coupled path. The equivalent coupler should have an optical gain, as show in Fig. S1(c), which would increase the magnitudes of the gain, loss and coupling coefficients contributed by the coupler by a factor of $\gamma_{0}$.

To analyze the coupling mechanism in a PT-symmetric system, one can write the coupling matrix as a summation of the lossless coupling part and a lossy part^1^,

$$F=\left[\begin{matrix} \gamma_{c} & i\kappa\\ i\kappa& \gamma_{c} \end{matrix}\right]+\left[\begin{matrix} \alpha_{1}+\alpha_{3}+\gamma_{0}-\gamma_{c} & \gamma_{0} \\ \gamma_{0} & \alpha_{1}+\alpha_{2}+\gamma_{0}-\gamma_{c} \end{matrix}\right]$$

where the first matrix is the transfer matrix of the lossless coupler, the second matrix is the loss and gain induced to by the polarizers and the EDFA, $\alpha_{1}$ and $\alpha_{2}$ are the loss coefficients introduced by PC1 to the two polarization modes, $\alpha_{3}$ is universal cavity loss introduced to the two polarization modes when PC2 is rotated. Note that Eq. is written as the summation of gain, loss and coupling coefficients because those factors are expressed in a per-time-unit fashion, in contrast to those in a per-round-trip fashion in Fig. S1. Energy conservation in a lossless coupler requires that $\gamma_{c}$ and $\kappa$ are real numbers and $\gamma_{c}^{2}+\kappa^{2}=1$ (here, $\gamma_{c}$ and $\kappa$ are expressed in a per-round-trip fashion)^2^. However, with the extra gain and loss introduced to the polarization modes (the second matrix on the right-hand side of Eq. ), the total transfer matrix $F$ does not satisfy energy conservation. It should be noted that $\gamma_{c}$ and $\kappa$ are real numbers even if there is an optical loss involved in the coupling process, which applies to coupling based on a reciprocal optical coupling component even with scattering and absorption losses^3^, including a polarizer-based coupling system we incorporated.

Based on the above discussion and Fig. S1, the coupled differential equations of the polarimetric PT-symmetric system can be written, which are given as Eqs. (9) and (10) in the main text.

**3. Polarization diversity gain/loss measurement**

The polarization-dependent gain and loss coefficients of the fiber loop is critical for the realization of PT-symmetric operation. An experimental verification is provided show the tuning of the gain and loss coefficients to achieve PT symmetry. To do this, we use a measurement setup shown in Fig. S2, in which an erbium-doped optical amplifier (EDFA) is incorporated to provide the gain. The EDFA is controlled to operate below saturation, so the output power is input power dependent. The laser loop is disconnected at the birefringence path between a polarization controller (PC1) and a polarizer (Pol. 1), and two linearly polarized light waves with different wavelengths from two external laser sources are combined at a polarization beam combiner (PBC) and launched into the loop via PC1. A second PC (PC2) is connected after the EDFA and second polarizer (Pol. 2) is connected after a tunable optical filter (TOF). After circulating for one round trip, the light waves are directed to an optical spectrum analyzer (OSA) which is used to monitor the optical spectrum and observe the optical power variations when the two PCs are tuned. The variations of the polarization-dependent gain and loss are consequently measured.

The measurement results are shown in Fig. S3. By tuning the half-wave plate in PC1 and PC2, we observed that round-trip gains of the *x* and *y* polarization modes change differently and uniformly, respectively, which agree well with our analysis.

**References**

1 Özdemir, Ş., Rotter, S., Nori, F. & Yang, L. Parity–time symmetry and exceptional points in photonics. *Nature Materials* **18**, 783-798 (2019).

2 Hui, R. & O'Sullivan, M. *Fiber optic measurement techniques*. (Academic Press, 2009).

3 McKinnon, W. *et al.* Extracting coupling and loss coefficients from a ring resonator. *Optics Express* **17**, 18971-18982 (2009).

**Fig. S1.** (a) A spatial PT-symmetric system equivalent to the polarimetric system we proposed. The polarizers would introduce mutual coupling between the two orthogonal polarization states and function equivalently to a lossy coupler. A lossless coupler in (b) and four attenuators (Att. 1, 2 and 3s) are used to mimic the operation of such a lossy coupler. In addition, the EDFA is in the coupled path of the polarimetric PT-symmetric laser would result in an equivalent coupler in (c) with the gain, loss and coupling coefficients increased by a factor of $\gamma_{0}$.


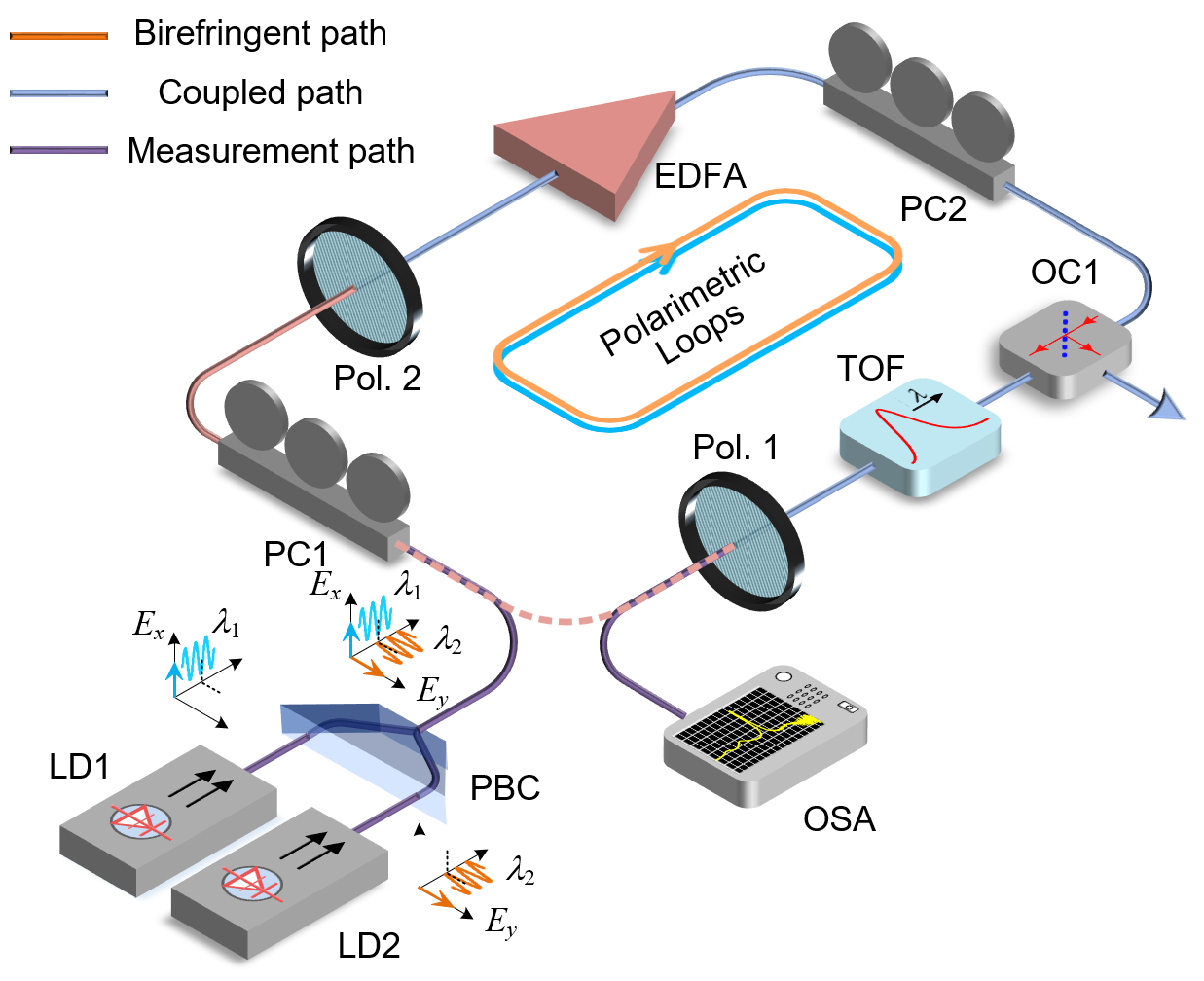


**Fig. S2. Setup for polarization mode gain and loss coefficient measurement**. Two linearly polarized light waves from two laser sources with slightly different wavelengths are combined at a polarization beam combiner (PBC) and launched into the laser cavity via PC1. The light waves after one round trip are directed to an optical spectrum analyzer (OSA). Since the polarization states of the two light waves at the output of the PBC are orthogonal, a polarization-dependent cavity loss can be analyzed by monitoring the power variation of each wavelength component.

**Fig. S3. Gain tuning in the two polarimetric loops by tuning PC1 and PC2.** (a) Optical spectra when PC1 is tuned to change the gains of the polarimetric loops; (b) the corresponding optical powers of the two wavelengths measured by the OSA; (c) optical spectra when PC2 is tuned to change the cavity loss of the two polarimetric loops; (d) the corresponding optical powers of the two wavelengths measured by the OSA.
